# Doxorubicin Incorporation into Gold Nanoparticles: An In Vivo Study of Its Effects on Cardiac Tissue in Rats

**DOI:** 10.3390/nano14201647

**Published:** 2024-10-14

**Authors:** Patricia Lorena Dulf, Camelia Alexandra Coadă, Adrian Florea, Remus Moldovan, Ioana Baldea, Daniel Vasile Dulf, Dan Blendea, Luminita David, Bianca Moldovan, Valentina Ioana Morosan, Sergiu Macavei, Gabriela Adriana Filip

**Affiliations:** 1 Faculty of Medicine, Iuliu Haţieganu University of Medicine and Pharmacy, 400012 Cluj-Napoca, Romania; cimpan.patricia@umfcluj.ro (P.L.D.); dulf.daniel@umfcluj.ro (D.V.D.); 2 Department of Molecular Sciences, Iuliu Haţieganu University of Medicine and Pharmacy, 400012 Cluj-Napoca, Romania; aflorea@umfcluj.ro; 3 Department of Functional Biosciences, Iuliu Haţieganu University of Medicine and Pharmacy, 400012 Cluj-Napoca, Romania; moldovan.remus@umfcluj.ro (R.M.); gabriela.filip@umfcluj.ro (G.A.F.); 4 Municipal Clinical Hospital, 400139 Cluj-Napoca, Romania; 5 Internal Medicine Department, Faculty of Medicine, Iuliu Haţieganu University of Medicine and Pharmacy, 400012 Cluj-Napoca, Romania; dblendea@me.com; 6 Department of Cardiology, Heart Institute, 400001 Cluj-Napoca, Romania; 7 Research Centre for Advanced Chemical Analysis, Instrumentation and Chemometrics, Faculty of Chemistry and Chemical Engineering, Babes-Bolyai University, 400347 Cluj-Napoca, Romania; luminita.david@ubbcluj.ro (L.D.); bianca.moldovan@ubbcluj.ro (B.M.); valentina.morosan@ubbcluj.ro (V.I.M.); 8 National Institute for Research and Development of Isotopic and Molecular Technologies, 400293 Cluj-Napoca, Romania; sergiu.macavei@itim-cj.ro

**Keywords:** oxidative stress, cardiotoxicity, antioxidants, doxorubicin, metal nanoparticles, antineoplastic agents

## Abstract

Gold nanoparticles (Au-NPs) have been explored as potential vectors for enhancing the antitumor efficacy of doxorubicin (DOX) while minimizing its cardiotoxic effects. However, the impacts of DOX Au-NPs on cardiac function and oxidative stress remain inadequately understood. This study aimed to explore the effects of DOX Au-NPs in comparison to free DOX, focusing on oxidative stress markers, inflammation, ultrastructural changes, and cardiac function. Male rats were divided into the following four groups: control, citrate Au-NPs, DOX, and DOX Au-NPs. Cardiac function was assessed using echocardiography, and oxidative stress was evaluated through Nrf2, malondialdehyde (MDA) and superoxide dismutase (SOD) levels, and the GSH/GSSG ratio. The ultrastructure of cardiac tissue was assessed by transmission electron microscopy (TEM). Rats treated with DOX Au-NPs exhibited significant cardiac dysfunction, as indicated by a reduction in fractional shortening and ejection fraction. Oxidative stress markers, including elevated MDA levels and a reduced GSH/GSSG ratio, were significantly worse in the DOX Au-NP group. SOD levels decreased, indicating compromised antioxidant defenses. Citrate Au-NPs also caused some alterations in cardiac function and ultrastructure but without other molecular alterations. DOX Au-NPs failed to mitigate cardiotoxicity, instead exacerbating oxidative stress and cardiac dysfunction. DOX Au-NPs possess cardiotoxic effects, necessitating further investigation into alternative nanoparticle formulations or therapeutic combinations to ensure both efficacy and safety in cancer treatment.

## 1. Introduction

Chemotherapy has revolutionized cancer treatment over the past five decades, with drugs like doxorubicin (DOX), an anthracycline, playing a central role in the management of a wide range of malignancies, such as leukemia, lymphoma, breast cancer, and osteosarcoma. DOX works by intercalating into DNA, inhibiting topoisomerase II, and generating free radicals that cause DNA damage and trigger apoptosis in rapidly dividing cells [1]. Despite its effectiveness, DOX lacks selectivity, with only 0.1–1% of the drug reaching tumor cells, while the remaining drug affects healthy tissues, particularly the heart, leading to significant cardiotoxicity [2,3,4,5].

DOX cardiotoxicity is dose-dependent, with higher cumulative doses increasing the risk of left ventricular dysfunction and heart failure. Acute cardiotoxicity can manifest as arrhythmias or myocarditis, while chronic effects, including a progressive decline in left ventricular ejection fraction, are more common and can lead to symptomatic heart failure [5]. In pediatric patients, around 10% develop heart failure after completing DOX-based chemotherapy. These cardiac complications often necessitate the interruption or cessation of cancer treatment, negatively impacting patient survival and prognosis [6,7].

To address the issue of DOX-induced cardiotoxicity, researchers have explored various strategies aimed at reducing off-target effects, including optimizing dosing regimens, employing advanced drug delivery systems and administration of drugs with cardioprotective effects [8]. One promising strategy involves utilizing gold nanoparticles (Au-NPs) as carriers for targeted DOX delivery to tumor cells. Recent advances in nanomedicine have showed that Au-NPs can enhance drug accumulation within malignant tissues through the enhanced permeability and retention effect, which allows nanoparticles to preferentially localize in tumors due to abnormal vascular permeability and impaired lymphatic drainage. Au-NPs are considered inert metallic nanoparticles with many advantages due to their ability to release in tumor cells of a wide variety of molecules including DNA, siRNA, chemotherapeutic agents and proteins [9]. Moreover, Au-NPs can be functionalized with specific ligands, such as antibodies or peptides, to enable highly selective targeting of tumor cells, thus reducing the exposure of healthy tissues to the cytotoxic effects of DOX [10,11]. Currently, the majority of existing literature focuses on the combination of DOX with other elements such as polyethylene glycol (PEG) rather than the direct binding of DOX to Au-NPs.

Evidence suggests that DOX-Au-NPs can improve the delivery of DOX to cancer cells while reducing systemic toxicity [9,12,13], including cardiotoxic effects. However, optimizing the size, shape, and surface chemistry of Au-NPs is essential to achieve maximal therapeutic efficacy and minimize toxicity [14,15]. Functionalization of Au-NPs with tumor-specific ligands can possibly enhance the selectivity of drug delivery, thereby minimizing the risk of adverse effects on non-cancerous tissues [16].

This study aimed to evaluate the impact of DOX-functionalized Au-NPs (DOX Au-NPs) on cardiac tissue, focusing on oxidative stress, inflammation, and cardiac function. The findings were compared to free DOX and citrate-reduced Au-NPs. The goal was to explore whether DOX Au-NPs can exert antitumor efficacy while minimizing cardiotoxic effects, offering a safer and more effective cancer treatment strategy.

## 2. Materials and Methods

### 2.1. Preparation and Characterization of Gold Nanoparticles

#### 2.1.1. Synthesis of Au-NPS+Citrate

Gold nanoparticles were produced by reducing HAuCl_4_ with trisodium citrate. The synthesis was carried out as follows: 50 mL of 1 mM HAuCl_4_ solution was heated until boiling under constant stirring. Once the HauCl_4_ solution started boiling, 5 mL of 38.8 mM trisodium citrate solution was added quickly to the boiling solution. The solution was then heated until the color changed from pale yellow to a wine-red color specific to the formation of the gold nanoparticles. After the appearance of the wine-red color, the solution was removed from heat and left to cool. The formation of citrate Au-NPs was confirmed using UV-Vis spectroscopy.

#### 2.1.2. Synthesis of Doxorubicin-Functionalized Nanoparticles (DOX Au-NPs)

The synthesis of DOX Au-NPs was carried out by displacing citrate from the surface of Au-NPs by the DOX molecule. For this purpose, 4 mL of 15.3 mg Au/mL citrate Au-NPs was mixed with 0.4 mL of 35 μg/mL DOX. The obtained mixture was then left to react at room temperature for 24 h. At the end of this time interval, the color of the solution changed from a wine-red color specific to citrate Au-NPs to a violet–blue color specific to DOX Au-NPs. The DOX Au-NP solution obtained was centrifuged at 10,000 rpm for 40 min, the supernatant was removed, and the precipitate was resuspended in distilled water. The purified nanoparticles were analyzed using adequate analysis methods.

The binding of DOX on the surface of the nanoparticles was determined spectrophotometrically by determining the concentration of DOX found in the centrifuged supernatant [17,18].

The binding degree was calculated using the following formula:

$$binding\;degree\;\%=\frac{q_{doxo\;i}-q_{doxo\;s}}{q_{doxo\;i}}*100$$

where *binding degree %* —the percentile of doxorubicin bounded to the surface of AuNPs (%); *q_doxo i_*—the initial quantity of doxorubicin (mg/mL); and *q_doxo s_*—the quantity of doxorubicin in supernatant (mg/mL).

#### 2.1.3. Characterization of the DOX-Functionalized Gold Nanoparticles (DOX Au-NPs)

The UV-Vis spectroscopy technique was initially used to monitor the synthesis of both citrate Au-NPs and DOX Au-NPS nanoparticles. UV-Vis spectra were obtained using a Perkin Elmer Lambda 25 spectrometer (Perkin Elmer, Shelton, CT, USA), scanning the absorbance between 300 and 800 nm in 1 cm quartz cuvette, using distilled water as a blank. The doxorubicin-functionalized gold nanoparticles were characterized by transmission electron microscopy (TEM) using a Hitachi H-7650 automatic transmission electron microscope (HHT, Tokyo, Japan) at 120 kV on a carbon-coated copper grid. The mean diameter of the synthesized DOX Au-NPs was determined using ImageJ 1.53m software, based on at least 40 nanoparticles. The presence of the doxorubicin on the surface of the Au-NPs was confirmed by Fourier transform infrared spectroscopy (FTIR), using a Bruker Vector 22 FTIR spectrometer (Brucker, Rosenheim, Germany), in a KBr pellet, in the range of 4000–500 cm^−1^. The crystalline structure of the synthesized DOX Au-NPs was investigated by X ray spectroscopy using a Smart Lab Rigaku diffractometer (Rigaku, Tokyo, Japan) with a graphite monochromator with Cu-Ka radiation (k ¼ 1:54 Å); X-ray source: anode Cu, 9 kW. The hydrodynamic diameter and zeta potential of the synthetized DOX Au-NPs were evaluated using a Zetasizer Nanoseries compact scattering spectrometer (Malvern Instruments Ltd., Malvern, UK) in order to determine the stability of the synthesized nanoparticles.

### 2.2. Biological Effects of the Dox Au-NPs Experimental Design

**Figure 1:**
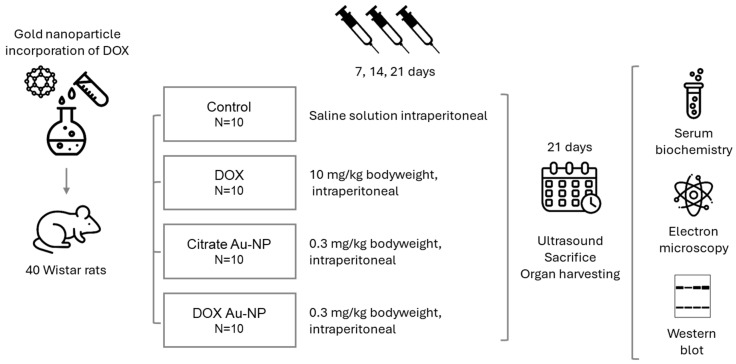
Study workflow: Rats were divided into the following four groups: control, DOX, citrate Au-NP, and DOX Au-NP. After a period of 21 days of treatment, cardiac ultrasound was performed, and the animals were euthanized with the harvesting of samples and organs for subsequent analyses.

This was an in vivo study conducted on 40 male Wistar rats, aged 3 months and with an average weight of 230 ± 20 g, which were randomly divided into the following four groups: control, DOX, citrate Au-NP, and DOX Au-NP. Treatments were administered as follows: the control group received saline solution; DOX group received DOX at a dose of 10 mg/kg body weight; citrate Au-NP group received the control citrate Au-NP at a dose of 0.3 mg/kg bodyweight; and DOX Au-NP animals received the same dose of 0.3 mg/kg bodyweight of DOX-coated Au-NP (Figure 1). All doses were administered intraperitoneally on days 7, 14, and 21. After 21 days of treatment, according to the aforementioned administration schemes, cardiac ultrasound examinations were performed, and the animals were euthanized with an overdose of anesthetic cocktail (2.5 mg/100 g b.w. ketamine 10% and 50 mg/100 g b.w. xylazine hydroxychloride 2%). Tissues and blood samples were collected for subsequent analysis.

### 2.3. Rat Cardiac Ultrasound

Cardiac ultrasound (US) investigations were performed with an Ultrasonix ultrasound machine with a 15–20 Hz probe for rats with the following settings: transmission frequency of 15 MHz, a depth of 1.5 cm, and a frame rate of 25 frames per second as previously described [4,8]. Briefly, the rats were anesthetized with standard doses of ketamine and xylazine, and the measurements were conducted in the M-mode. The left ventricular ejection fraction (LVEF) was determined using the Teicholz method [19]. A minimum of four measurements were performed for each rat, and the results were averaged. All measurements adhered to the American Society of Echocardiography’s guidelines [20].

### 2.4. Biochemical and Western Blot Assays

Oxidative stress parameters from the cardiac tissues of the four study groups were assessed through the measurement of malondialdehyde (MDA) as a marker of lipid peroxidation, reduced glutathione (GSH), oxidized glutathione (GSSG), and the ratio GSH/GSSG and SOD activity. SOD activity was measured using the cytochrome *c* reduction test [21]. The analyzed tissues were homogenized with a Polytron homogenizer using Tris buffer solution and the protein levels were measured with the Bradford method (Sigma-Aldrich Chemicals GmbH, Burlington, MA, USA).

Nrf2 and pNFkB protein levels in cardiac tissues were evaluated by Western blot. A total of 40 µg/lane of each sample was loaded on SDS-PAGE gels and subsequently transferred to PVDF membranes (Bio-Rad Mini-protean system). Blocking was conducted with StartingBlock Buffer (Thermo Fisher Scientific) and then incubated with primary antibodies obtained from Santa Cruz Biotechnology INC (Dallas, TX, USA), targeting p-NFkB (p68 sc-136548 mouse monoclonal) and Nrf2 (437C2a: sc-81342). Membranes were washed and then incubated with an anti-mouse secondary antibody linked with HRP (#7074, CellSignaling Technology, Danvers, MA, USA) for 90 min at room temperature. Supersignal West Femto-Chemiluminescent substrate (Thermo Fisher Scientific, Rockford, IL, USA) was used for the detection step and image acquisition was performed on a ChemiDoc System (Bio-Rad, Hercules, CA, USA). Band quantification was performed using the Image Lab version 6.1.0 built 7 analysis software (Bio-Rad, Hercules, CA, USA). Beta-actin was used for normalization.

### 2.5. Transmission Electron Microscopy

Samples of approx. 2 mm^3^, collected from the left ventricular myocardium, were prefixed 2 h with 2.7% glutaraldehyde (Agar Scientific, Stansted, UK) in 0.1 M phosphate buffer (pH = 7.4), washed 4 times (3 × 1 h, 1 overnight) with the same buffer, and postfixed 1.5 h with 1% OsO4 (Electron Microscopy Sciences, Hatfield, PA, USA) in 0.15 M phosphate buffer (pH = 7.4). They were then dehydrated with an acetone series (Merck, Darmstadt, Germany) (30–100%, 15–30 min each bath) and infiltrated with an EMBED 812 epoxy resin series (Electron Microscopy Sciences, Hatfield, PA, USA) in acetone (30–90%, 1 h each, and pure resin overnight). The resin was polymerized for 72 h at 60 °C. Ultrathin sections of 70–80 nm were cut with an ultra 45° diamond knife (DiATOME AG, Nidau, Switzerland) on a Bromma 8800 ULTRATOME III ultramicrotome (LKB, Stockholm, Sweden), collected on 300 mesh copper grids (Agar Scientific, Stansted, UK), and covered with a formvar (Electron Microscopy Sciences, Hatfield, PA, USA) film. The sections were double contrasted, as follows: 15 min with 13% uranyl acetate (Merck, Darmstadt, Germany) and 5 min with 2.8% lead citrate (Fluka, Buchs, Switzerland). Then, they were examined with a JEOL JEM 100CX II transmission electron microscope (JEOL, Tokyo, Japan) at 80 kV. Images were recorded with a MegaView G3 camera and Radius 2.1 software (both from EMSIS GmbH, Münster, Germany).

### 2.6. Statistical Analysis

The statistical analysis was conducted using R version 4.4.0 (2024-04-24 ucrt)-“Puppy Cup” [22]. Data are presented through box plot graphs with the length of the whiskers set to a 1.5 multiple of the interquartile range. The normality of the data distribution was tested for all variables using the Shapiro–Wilk test. Based on the type of distribution, differences among groups were tested using either ANOVA or Kruskal–Wallis tests as appropriate. To further evaluate which of the groups carry a statistical significance, post hoc tests were conducted, and all *p*-values were corrected for multiple comparisons. A *p*-value of 0.05 was set as a threshold for statistical significance.

## 3. Results

### 3.1. Characterization of Gold Nanoparticles

#### 3.1.1. UV-Vis Spectroscopy

**Figure 2:**
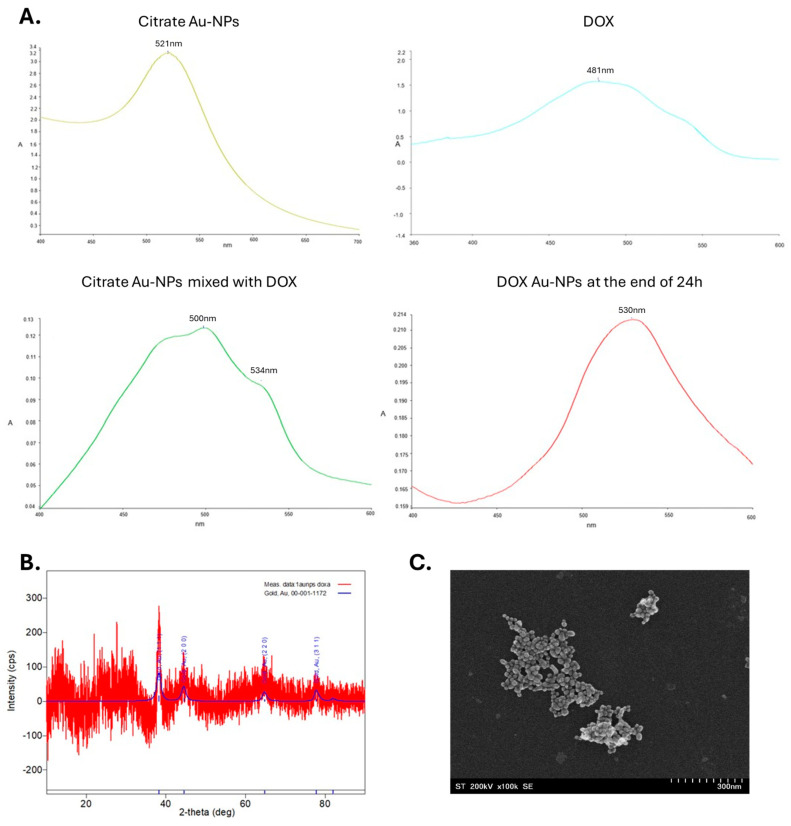
(**A**) UV-Vis spectra of citrate Au-NPs, DOX, citrate Au-NPs mixed with DOX at T_0_, and DOX Au-NPs after 24 h; (**B**) XRD spectrum of DOX Au-NPs; (**C**) TEM image of DOX Au-NPs.

UV-Vis spectroscopy was used to confirm the synthesis of the nanoparticles. Metal nanoparticles exhibit unique optical properties due to the resonance of surface plasmons (SPRs). For gold nanoparticles, the excitation of SPRs in a colloidal solution, results in an absorption maximum at wavelengths ranging from 500 to 550 nm [23]. In the case of citrate Au-NPs after the synthesis (Figure 2A), an absorption maximum at 521 nm appeared, which confirmed the formation of the gold nanoparticles.

When comparing the spectra of citrate Au-NPs and DOX with the spectrum of the mixture of Au-NPs and DOX at T_0_ one can observe a shift in the absorption maxima for DOX from 481 nm to 500 nm and for Au-NPs from 521 nm to 534 nm. This shift confirms interactions between the nanoparticles and DOX and the conjugation of the DOX with the gold nanoparticles. After 24 h of incubation the absorption maximum of DOX was no longer visible, instead a broad band between 500 and 550 nm with a maximum of 530 nm appeared, confirming the formation of the DOX Au-NPs (Figure 2A). The bonding degree determined from the centrifugation supernatant was 89% ± 2% at the end of the 24 h.

#### 3.1.2. XRD DOX Au-NPs

The crystalline structure of the synthetized DOX nanoparticles was investigated using X-ray spectroscopy. In Figure 2B, the XRD spectrum clearly reveals the formation of crystalline gold, as indicated by the presence of four distinct diffraction peaks at 2Ɵ values of 38.23; 44.64; 64.83, and 77.8° corresponding to the (1,1,1), (2,0,0), (2,2,0), and (3,1,1) planes of the cubic crystal of gold, according to JCPDS 00-001-1172.

#### 3.1.3. TEM of DOX Au-NPs

The morphology and shape of the doxorubicin-functionalized gold nanoparticles were analyzed using transmission electron microscopy (TEM). The TEM image (Figure 2C) suggests that the synthesized nanoparticles are polydisperse and spherical in shape with an average diameter of 22.68 ± 8.44 nm.

#### 3.1.4. FTIR Analysis

**Figure 3:**
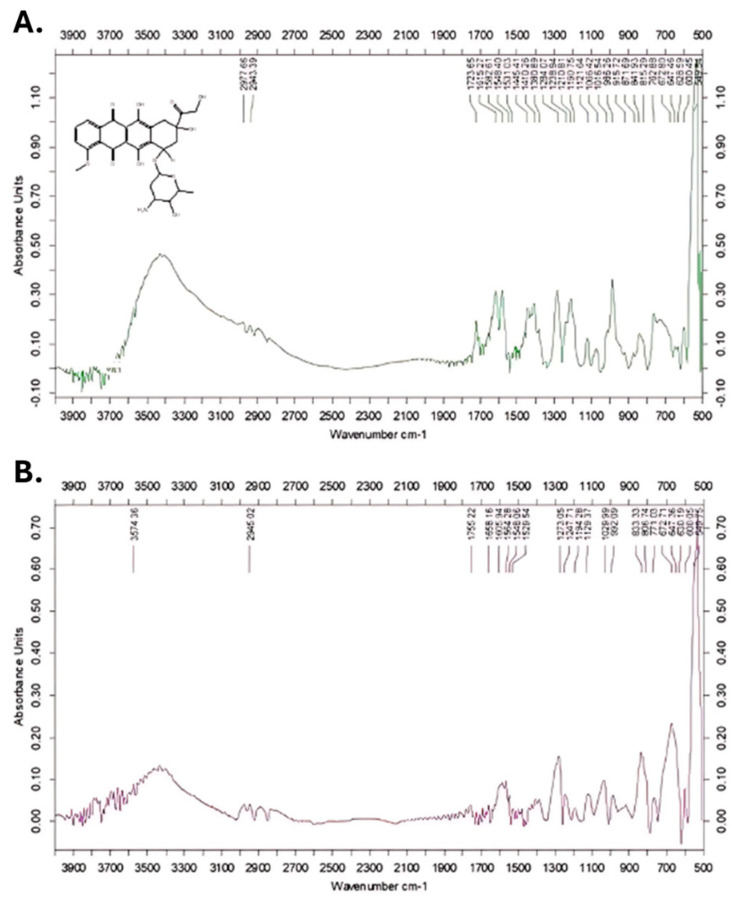
(**A**) FTIR spectra of DOX; (**B**) FTIR spectra of DOX Au-NPs.

An FTIR analysis was conducted to identify and confirm the presence of DOX on the surface of the metal nanoparticles. The FTIR spectrum of DOX (Figure 3A) displayed a broad absorption band between 3200 and 3600 cm^−1^, characteristic of the OH group vibrations in DOX. The peak at around 1723 cm^−1^ corresponds to the vibration of the C=O bond in the anthracycline structure from DOX. In the FTIR spectrum of the DOX Au-NPs (Figure 3B), similar absorption bands were observed, as follows: a broad peak at 3574 cm^−1^ corresponding to OH group vibrations; a band at 2945 cm^−1^, attributed to the stretching vibration of the C-H bond; and a peak at 1755 cm^−1^, indicating the presence of the C=O bond in the biomolecules located on the surface of the metal nanoparticles. These absorption bands confirm the presence of DOX molecules on the surface of synthesized metallic nanoparticles.

#### 3.1.5. Stability of Au-NPs+DOX

**Figure 4:**
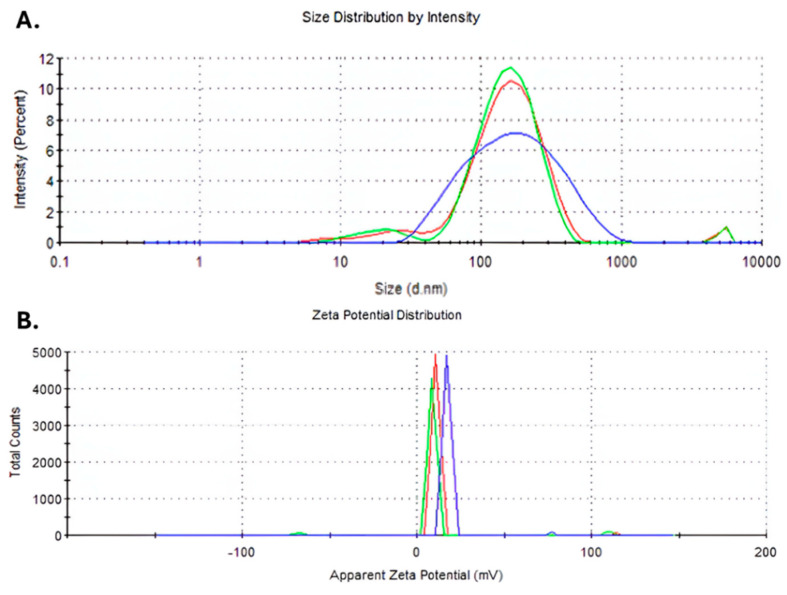
(**A**) Hydrodynamic diameter; (**B**) zeta potential. Blue, red and green lines represent three different replicates.

The stability of the synthesized nanoparticles can be estimated through the value of the zeta potential. The hydrodynamic diameter had a value of 118.6 nm (Figure 4A). The measurements indicated a value of the zeta potential of 17.3 mV, which shows a low stability for the DOX-functionalized gold nanoparticles (Figure 4B). The determined PDI index of the sample was 0.44.

#### 3.1.6. DOX Au-NPs’ Effect on Cardiac Output

**Figure 5:**
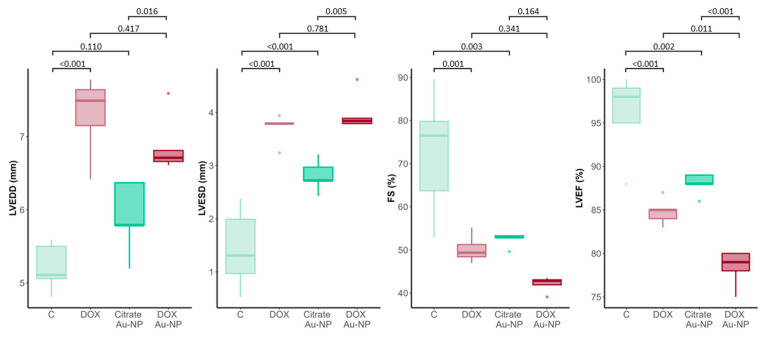
Cardiac ultrasound parameters in the four treatment groups. N = 5 samples analyzed for each group. C: control; DOX: doxorubicin; citrate Au-NP: citrate gold nanoparticle; Au-NP: gold nanoparticle; LVEDD: left ventricular end-diastolic diameter; LVESD: left ventricular end-systolic dimension; FS: fractional shortening; LVEF: left ventricular ejection fraction.

We sought to evaluate whether the molecular and ultrastructural changes seen in animals treated with DOX Au-NPs resulted in anatomical and functional damage, as measured by cardiac ultrasound. Both the left ventricular end-diastolic diameter and left ventricular end-systolic dimension were significantly larger in the DOX-treated animals (*p* < 0.001 for both groups). Similarly, DOX Au-NPs exerted a detrimental effect on cardiac tissue, with both left ventricular end-diastolic diameter and left ventricular end-systolic dimension being significantly increased vs. the citrate Au-NP control (*p* = 0.016 and *p* = 0.005, respectively). When comparing the DOX Au-NP-treated animals with those receiving standard DOX, no differences were seen in these parameters. Moreover, this effect was also seen in the case of citrate Au-NPs, with the enlargement of the left ventricular end-systolic dimension reaching statistical significance (*p* < 0.001). As far as the cardiac function expressed through the ejection fraction is concerned, a significant decrease was seen in the groups receiving DOX and DOX Au-NPs (*p* < 0.001 for both). Moreover, in the DOX Au-NP group, the ejection fraction values were significantly lower than those seen in the DOX group (*p* = 0.011) (Figure 5).

### 3.2. DOX-Coated Gold Nanoparticles’ Effects on Oxidative Stress

**Figure 6:**
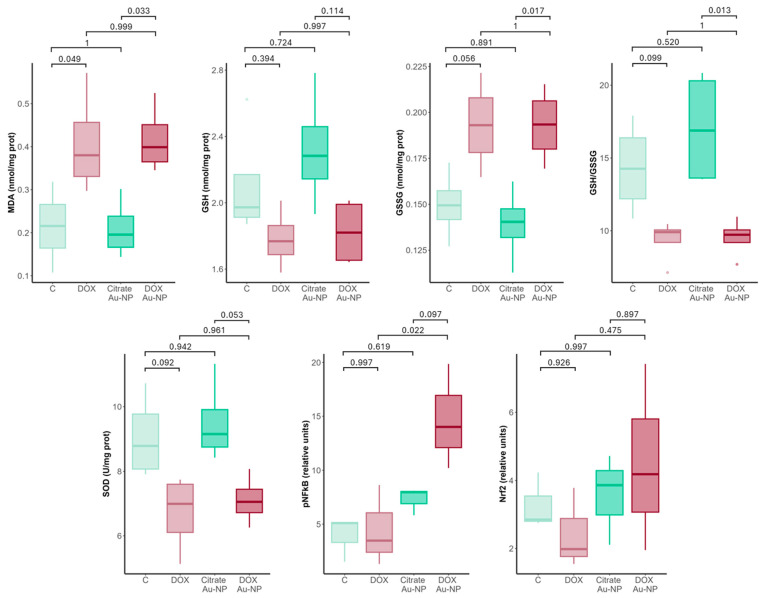
Oxidative stress and antioxidant defense system levels in cardiac tissue homogenates in the four study groups. N = 3 samples analyzed for each group for pNFkB and Nrf2; N = 4 samples analyzed for MDA, SOD, GSH, and GSSG. MDA, GSH, and GSSG were determined from serum samples, while SOD was determined from lysed erythrocytes. C: control; DOX: doxorubicin; citrate Au-NP: citrate gold nanoparticle; Au-NP: gold nanoparticle; MDA: malondialdehyde; GSH: glutathione; GSSG: glutathione disulfide.

We sought to explore the effect of DOX and DOX Au-NP administration on the levels of oxidative damage and antioxidant response. We measured MDA as a marker of increased oxygen radical activity [24], GSH and GSSG for the assessment of toxicological responses and indicators of antioxidant activity [25]. Comparison of the cardiac tissues from DOX-treated animals showed increased levels of MDA and an imbalance in GSSG and the GSH/GSSG ratio of borderline significance (*p* = 0.056 and *p* = 0.099, respectively). Similarly, SOD levels decreased in the DOX-treated group, without reaching significance after multiple comparison correction (*p* = 0.092) (Figure 6).

As far as the effect of DOX Au-NPs on cardiac oxidative stress, the results show significant alterations in multiple of the analyzed parameters. Namely, MDA was significantly increased with respect to the citrate Au-NP control (*p* = 0.033), a level similar to that seen in DOX-treated rats (*p* = 0.999). GSSG and the resulting GSH/GSSG ratio were also significantly affected (*p* = 0.017 and *p* = 0.013, respectively) (Figure 6). SOD levels were also depleted in the DOX Au-NP-treated animals, but with a *p*-value at the limit of significance (*p* = 0.053). Gold nanoparticles prepared with citrate diminished the lipid peroxidation and increased GSH levels and the GSH/GSSG ratios, as well as improved SOD activity. Regarding the signaling pathways related to the oxidative stress response and inflammation, the pNFkB and Nrf2 levels were analyzed in the four study groups. While a degree of alteration was seen both in the DOX and DOX Au-NP-treated rats, only the pNFkB levels differed significantly among the analyzed groups. Namely, the DOX Au-NP animals showed increased levels of pNFkB with respect to the DOX treatment (*p* = 0.022). Moreover, these levels also tendentially increased in the DOX Au-NP animals with respect to the citrate Au-NP controls (*p* = 0.097) (Figure 6).

### 3.3. Ultrastructural Analysis of the Cardiac Tissues

**Figure 7:**
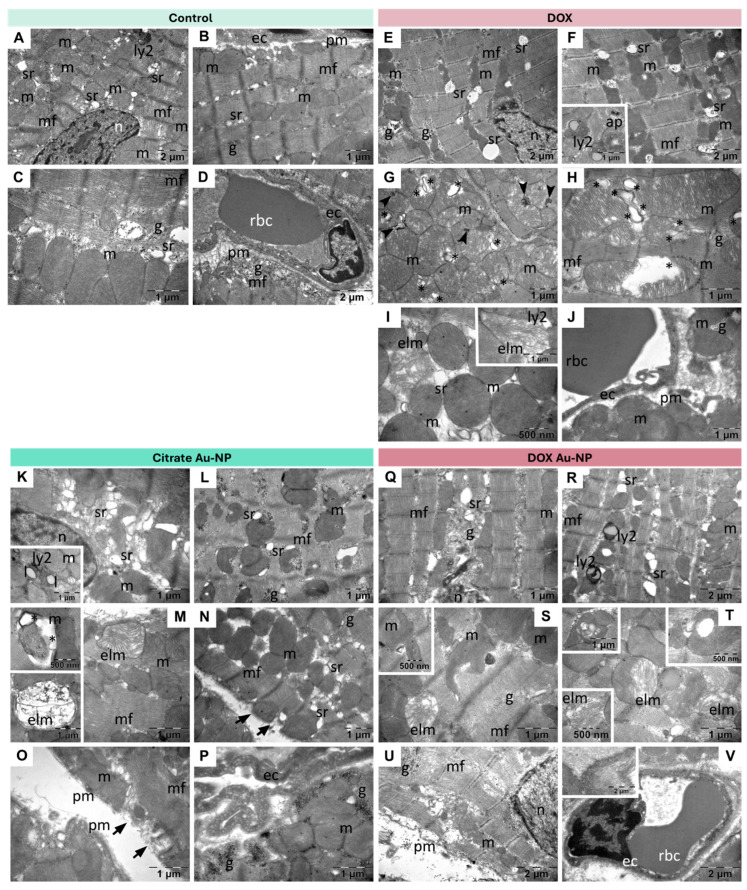
Representative TEM images of cardiac tissues from the four study groups. (**A**–**D**) Control: normal ultrastructural aspect of the myocardium samples from the control group. (**E**–**J**) DOX: extensive ultrastructural alterations of mitochondria and sarcoplasmic reticulum in the myocardium samples from the DOX group; asterisk: mitochondrial whorls of membranes. (**K**–**P**) Citrate Au-NP: diverse ultrastructural alterations in the myocardium samples from the citrate Au-NP group. arrow: disrupted plasma membrane; arrowhead: abnormal mitochondrial cristae with multiple membranes; asterisk: mitochondrial whorls of membranes; (**Q**–**V**) DOX Au-NP: limited ultrastructural alterations of mitochondria and sarcoplasmic reticulum in the myocardium samples from the DOX Au-NP group. ec: endothelial cell; g: glycogen; ly2: secondary lysosome; m: mitochondria; mf: myofibrils; n: nucleus; pm: plasma membrane; rbc: red blood cell; sr: sarcoplasmic reticulum; ap: autophagosome; arrowhead: abnormal mitochondrial cristae with multiple membranes; elm: electron lucent mitochondrion; ly2: secondary lysosome; l: lipid droplet.

The TEM examination of the myocardium samples from control group revealed normal architecture for the muscular tissue, nuclei, and mitochondria (Figure 7A–C), as well as glycogen granules (Figure 7B,D). Blood capillaries had normal ultrastructures, with the thin endothelium containing a moderate number of transcytosis vesicles (Figure 7B,D).

In the DOX group, the cardiomyocytes showed normal myofibrils and nuclei but with enlarged endoplasmic reticula, abnormal mitochondria (Figure 7E–H), and numerous secondary lysosomes (inset of Figure 7F). A low number of mitochondria had an electron lucent matrix and shorter cristae (Figure 7I) with both membranes disrupted (inset of Figure 7I). A small number of transcytosis vesicles were identified in the capillaries (Figure 7J).

In the citrate Au-NP group, the endoplasmic reticulum was proliferated and/or enlarged (Figure 7K–M), with many secondary lysosomes and lipid droplets (inset of Figure 7K). Rare mitochondria were observed with normal cristae within an electron lucent matrix (Figure 7M) and with collapsed cristae (upper inset of Figure 7M), or they were entirely devoid of cristae and with disrupted membranes (lower inset of Figure 7M). In some regions of the tissue, the cytoplasm of the cardiomyocytes was rarefied (Figure 7N), and in such regions the plasma membrane was interrupted (Figure 7N,O). Many transcytosis vesicles were found in the capillaries (Figure 7P).

Lastly, in the DOX Au-NP group, the myofibrils from cardiomyocytes mostly preserved their normal ultrastructures (Figure 7Q,R), while the perinuclear spaces were expanded (Figure 7Q), and the endoplasmic reticula were enlarged (Figure 7Q,R). Numerous secondary lysosomes (Figure 7R,S) and autophagosomes (Figure 7S) were seen, as well as altered mitochondria (<20%) (Figure 7S,T), some with disrupted membranes (Figure 7T and lower inset in this figure). Other mitochondria preserved a generally normal aspect but contained black whorls of membranes (Figure 7Q), multiple abnormal vesicular cristae (left upper inset of Figure 7T), or even electron lucent large vesicular cristae (right upper inset of Figure 7T). The plasma membranes of several cells were interrupted with disorganized myofibrils (Figure 7U). Transcytosis vesicles were identified in the capillaries (Figure 7V). A detailed description of the alterations can be found in the Supplementary results.

## 4. Discussion

### 4.1. DOX Au-NPs Alter the Cardiac Tissue at a Molecular Level

In this study, we sought to provide a detailed assessment of the effects of DOX Au-NPs, with particular emphasis on key aspects of oxidative stress, myocardial inflammation, and ultrastructure, as well as the most relevant aspect, that of cardiac function. To elucidate the extent of redox imbalance, we measured MDA concentrations, a well-recognized indicator of lipid peroxidation and oxidative cellular injury. Additionally, the ratio of reduced GSH to oxidized GSSG was examined, serving as a critical marker of intracellular antioxidant capacity and redox homeostasis. Furthermore, we analyzed the activation of key signaling pathways implicated in oxidative stress and inflammation by quantifying the expression levels of pNF-kB (nuclear factor kappa-light-chain-enhancer of activated B cells) and Nrf2 (nuclear factor erythroid 2-related factor 2) [26,27]. NF-kB is a central regulator of inflammatory and oxidative processes, with its activation being associated with a range of pathological conditions, including doxorubicin-induced cardiotoxicity. NF-kB serves as a regulator of inflammatory and oxidative responses, with its activation linked to various pathologies, including DOX-induced cardiotoxicity. In contrast, Nrf2 plays an important role in cellular defense against oxidative stress by regulating the expressions of multiple antioxidant genes. Additionally, we evaluated the activity of SOD, a key enzyme in the neutralization of reactive oxygen species (ROS) generated during oxidative stress. SOD represents the myocardium’s primary defense mechanism against oxidative stress, converting the highly reactive superoxide anion into a less reactive species, hydrogen peroxide, which is subsequently cleared through other intracellular pathways [28].

The results of this study reveal a toxicological profile for the combination of DOX and gold nanoparticles (DOX Au-NPs) that closely resembles the toxicity observed with free DOX treatment. This similarity in toxicity was seen in the significantly elevated MDA levels, and in the reduction in the GSH/GSSG ratio, indicating a severe disruption in myocardial redox homeostasis. These findings suggest that DOX Au-NPs exert considerable oxidative stress on cardiac tissue, contributing to significant myocardial damage. In addition, the marked reduction in SOD activity, suggests a compromised antioxidant defense and thus, an increased susceptibility of the cardiac tissue to ROS-mediated damage, which likely contributes to the cardiotoxic effects observed. These pathological changes are further reflected in the evaluation of signaling pathways associated with oxidative stress and inflammatory responses, particularly those involving NF-kB and Nrf2. In fact, the pNFkB levels were significantly higher in the DOX Au-NPs than for DOX.

In summary, these findings emphasize that although gold nanoparticles hold potential as targeted delivery vectors for doxorubicin in antineoplastic therapy, this particular compound does not provide the necessary cardioprotective effects. On the contrary, the DOX Au-NPs had the same degree of alterations, if not worse than DOX alone. This observation highlights the need for further investigation into alternative synthesis techniques or functionalization strategies to minimize the negative impact on cardiac tissue while maintaining the antitumor efficacy of doxorubicin.

### 4.2. DOX Au-NPs’ Cardiotoxicity as Revealed by the Detrimental Impact on Cardiac Output

Lastly, cardiac function was evaluated across the four experimental groups using echocardiography, a non-invasive method for assessing cardiac performance. The findings showed the detrimental effects of DOX Au-NPs on cardiac function, revealing significant impairments in the echocardiographic parameters measured. Specifically, the left ventricular end-diastolic diameter, left ventricular end-systolic diameter, and the ejection fraction were significantly altered in the group treated with DOX Au-NPs. Additionally, the group treated with free DOX also exhibited significant alterations in all functional parameters analyzed, further confirming the well-documented cardiotoxicity of this chemotherapeutic agent.

An interesting finding was that of the effects of the control citrate Au-NPs. The results show that these nanoparticles did not induce significant molecular alterations in the cardiac tissue. The analysis of oxidative stress markers, such as the MDA levels and GSH/GSSG ratio, revealed values comparable to those observed in the control group treated with saline solution. This suggests that, in the absence of DOX, these Au-NPs neither disrupt redox homeostasis nor contribute to oxidative damage in the myocardium. Nevertheless, at a functional level, both the fractional shortening and ejection fraction were significantly reduced in this group as well, suggesting some degree of alteration, most likely in other pathways. For instance, the ejection fraction was significantly reduced from an average of approximately 97% in the untreated control group to around 88% in the Au-NP-treated group. While this reduction may not seem drastic in absolute terms, it could have a substantial impact in oncologic patients undergoing multiple treatments, where even a slight decrease in cardiac function could negatively influence clinical outcomes and therapeutic success, particularly in the presence of pre-existing cardiovascular comorbidities.

### 4.3. DOX Au-NPs’ Structure in the Context of the Obtained Cardiac Effects

As far as the structure of the DOX Au-NPs are concerned, comprehensive physicochemical characterization of the administered nanoparticles demonstrated the successful conjugation of DOX to the gold nanoparticles. The hydrodynamic diameter was substantially larger than that of the gold nanoparticle core, indicating the binding of DOX to the nanoparticle surface. The stability of the DOX Au-NPs was further assessed by measuring the zeta potential, which, at a value of 17.3 mV, indicated significant instability of the compound and an increased likelihood of dissociation from the nanoparticle surface. This instability could result in the generation of more cardiotoxic intermediates, thus potentially explaining the exacerbated damaging effects seen in the group of animals treated with DOX Au-NPs. In fact, the most pronounced reduction in cardiac function among the experimental groups was observed in rats treated with DOX Au-NPs. In this group, FS decreased to just over 40%, in contrast to an average of 77% in the saline-treated control group, while LVEF was below 80%, significantly lower than the approximately 85% recorded in the DOX-only group. These results indicate that the combination of Au-NPs with DOX markedly exacerbates cardiac toxicity, potentiating the deleterious effects of DOX on the myocardium.

A study published in 2006 investigated the effects of Au-NPs and highlighted their pro-oxidant properties, as demonstrated by the induction of lipid peroxidation. The authors reported that 5 nm gold nanoparticles induced significantly higher levels of oxidative stress and cytotoxicity compared to larger nanoparticles [29]. It was shown that 5 nm nanoparticles catalyze the production of nitric oxide (NO) from endogenous S-nitroso adducts containing thiol groups in blood serum. Nitric oxide readily reacts with superoxide, generating peroxynitrite (ONOO-), which can interact with lipids, DNA, and proteins either through direct oxidative mechanisms or via indirect damage mediated by free radicals [30]. The enhanced generation of ROS may be attributed to the relatively large surface area of these small Au NPs.

There are several potential mechanisms underlying nanoparticle toxicity, including epithelial tissue damage [31], inflammation, and oxidative stress responses [15,32]. Nanoparticles are similar in size to certain biological molecules, such as proteins and nucleic acids. Many of these biomolecules consist of long macromolecular chains that are folded and structured through weak, cooperative interactions among side groups. Gold nanoparticles may penetrate these complex folded structures, potentially causing significant alterations at the molecular level. In rat hepatocytes exposed to 10 nm gold nanoparticles and, to a lesser extent, in those exposed to larger particles, an increase in steatosis was observed. This may be due to lipid peroxidation, which can damage the rough endoplasmic reticulum and lead to the detachment of cytoplasmic lipoproteins, suggesting abnormal lipid metabolism [33]. In cancer, Au-NPs tend to accumulate in tumor tissues because of the enhanced permeability and retention (EPR) effect, allowing for targeted drug delivery and reducing off-target toxicity [34]. However, in systemic inflammation, vascular permeability increases more broadly, potentially altering nanoparticle biodistribution and leading to unintended accumulation in inflamed tissues [35,36]. This could either enhance therapeutic effects in localized inflammation or increase side effects. Understanding how Au-NPs behave under these conditions is crucial for optimizing their use in treatments, ensuring both efficacy and safety.

The interaction of nanoparticles with biological systems is also influenced by their size. Smaller gold nanoparticles are capable of penetrating biomolecules, a phenomenon not possible for larger nanoparticles. Studies have shown that inhaled nanoparticles can enter the bloodstream and reach organs, such as the liver and heart, or blood cells [33]. A smaller nanoparticle size leads to a dramatic increase in the surface-area-to-volume ratio, resulting in a greater proportion of the substance’s molecules being exposed on the surface, thus enhancing its intrinsic toxicity. This may explain why smaller gold nanoparticles are generally more toxic than larger particles of the same insoluble material when compared on a mass dose basis [37].

### 4.4. Study Limitations and Future Directions

It is worth discussing that the standardization of the compounds was conducted in gold concentration, as we could not accurately measure the release of DOX in the tissues. This may result in a variability in pharmacokinetics and bioavailability of the compound and, thus, an influence on the cardiac output due to the Au-NPs properties. While the standardization based on the gold concentration allowed for a direct comparison between the citrate Au-NPs and the DOX Au-NP groups, it results in challenges when comparing their effects with the free DOX group. Future research should focus on the standardization of both gold and DOX concentrations to ensure a more direct comparison between free DOX and DOX Au-NPs.

Overall, the results of this study not only highlight the limitations of Au-NPs as vectors for DOX in providing cardioprotection but also raise concerns that the nanoparticles themselves may exert cardiotoxic effects, at least to some extent, potentially through mechanisms distinct from those associated with DOX-induced cardiotoxicity. Taking this into consideration, it becomes evident that further studies are required to clarify the mechanisms by which Au-NPs may affect cardiac function. Moreover, the development of therapeutic strategies to mitigate DOX-induced adverse effects while preserving its antineoplastic efficacy remains an ongoing task.

## 5. Conclusions

In conclusion, while combining gold nanoparticles with doxorubicin has shown potential in enhancing antitumor efficacy in various studies, our study, which focused on cardiac effects, reveals that this approach does not mitigate doxorubicin-induced cardiotoxicity. Significant alterations in oxidative stress and ultrasound alterations suggest that DOX Au-NPs do not offer cardiac protection. These findings emphasize the need for further research into alternative strategies that maximize doxorubicin’s efficacy while minimizing its harmful effects on healthy tissues, particularly the heart. Exploring other nanoparticle types, formulations, or therapeutic combinations may provide safer and more effective options for patients.

## Data Availability

The data presented in this study are available on request from the corresponding author.

## Supplementary Materials

The following supporting information can be downloaded at: https://www.mdpi.com/article/10.3390/nano14201647/s1.
