# Supplementary material for: Doxorubicin Incorporation into Gold Nanoparticles: An In Vivo Study of Its Effects on Cardiac Tissue in Rats
Source: Nanomaterials (Basel). 2024 Oct 14;14(20):1647. doi: 10.3390/nano14201647 (PMC11510282; doi:10.3390/nano14201647)
Supplement: Supplementary file 1 [file nanomaterials-14-01647-s001.zip › nanomaterials-3231904-supplementary.pdf]

Ultrastructural analysis of the cardiac tissues – detailed description

TEM examination of myocardium samples from control group revealed normal architecture of the muscular tissue. Cardiomyocytes were filled with myofibrils displaying regular pattern of sarcomeres; between them, rows of round or elongated mitochondria with numerous cristae could be seen (Figure 7A-C). Profiles of sarcoplasmic reticulum, as well as dispersed or grouped small granules of glycogen were scattered between the myofibrils (Figure 7A-C), the later ones being also visible at the periphery of the cell (Figure 7B, D). Elongated nuclei, mainly euchromatic were surrounded by cytoplasm contained round mitochondria, a few endoplasmic reticulum profiles, rare secondary lysosome and glycogen granules (Figure 7A). Blood capillaries had normal ultrastructure, with thin endothelium containing moderate number of transcytosis vesicles (Figure 7B, D).

In DOX group, the cardiomyocytes showed myofibrils that preserved their normal aspect of sarcomeres (Figure 7E, F). The nuclei were also not affected, but in the cytoplasm surrounding them (Figure 7E), as well as between the myofibrils (Figure 7F) the endoplasmic reticulum was enlarged. No visible change in the amount of glycogen was noted. However, many electron-lucent regions interrupting the rows of mitochondria contained smaller or larger numbers of glycogen granules (Figure 7E, F). Secondary lysosomes appeared more numerous (inset of Figure 7F) as compared to control. In this experimental group, and also among all groups, mitochondria were the most affected organelles, despite the fact that many of them (about 40-50%) showed normal ultrastructure. Thus, there were mitochondria with normal aspect of cristae but containing black whorls of membranes of various sizes, resembling myelin figures (Figure 7G). Moreover, the pattern of mitochondrial cristae was disturbed in many mitochondria; such abnormal cristae showed a tendency to become vesicular, more or less round, and with multiple membranes (Figure 7G, H), sometimes more than 3-4 in the same mitochondrion, and in some cases extremely large (Figure 7H). A low number of mitochondria, found mainly in rarefied regions of the cytoplasm, had electron lucent matrix and shorter cristae (Figure 7I). Apart from this particular feature, in rare mitochondria both membranes were disrupted (inset of Figure 7I). In this context, it remains possible that, due to the incidence of the sections, more such mitochondria with rarefied matrix could have disrupted membranes. In the capillaries with normal endothelium, a low number of transcytosis vesicles were identified (Figure 7J).

In Citrate Au-NPs group, the endoplasmic reticulum was proliferated and/or enlarged both in the cytoplasm surrounding the nuclei of the cardiomyocytes (Figure 7K) and between the myofibrils (Figure 7L, M). Many secondary lysosomes and lipid droplets were identified in the proximity of nuclei (inset of Figure 7K). Mitochondria with normal ultrastructure were prevalent. Rare mitochondria contained normal cristae within an electron lucent matrix (Figure 7M). Others had collapsed cristae on the same electron lucent matrix (upper inset of Figure 7M), forming vacuoles, or were entirely devoid of cristae and with disrupted membranes invaded by small electron dense granules (probably glycogen) (lower inset of Figure 7M). In some regions of the tissue, the cytoplasm of the cardiomyocytes was rarefied (Figure 7N), and in such regions the plasma membrane was interrupted (Figure 7N, O). In the capillaries with normal endothelium, we found a high number of transcytosis vesicles (Figure 7P).

In DOX Au-NP group, the myofibrils from cardiomyocytes mostly preserved their normal ultrastructure (Figure 7Q, R). At the level of nuclei, the perinuclear space was expanded (Figure 7Q), and the endoplasmic reticulum was enlarged as well, both in the cytoplasm surrounding the nucleus (Figure 7Q) and between the myofibrils (Figure 7R), giving a general aspect of rarefaction to the cells. Numerous secondary lysosomes (Figure 7R, S) and autophagosomes (Figure 7S) were found in the cells from this group. Mitochondria in the cells from this group displayed diverse ultrastructural alterations, but those alterations concerned only a limited number of mitochondria (estimated to less than 20%). Thus, we observed enlarged and polymorphous mitochondria (Figure 7S), mitochondria with fewer and disrupted cristae within an entirely rarefied matrix (Figure 7S, T) or in a distinctly visible rarefied part of the matrix (Figure 7T). Sometimes, such rarefied mitochondria showed disrupted outer and inner membranes (Figure 7T and lower inset of this figure). Other mitochondria preserved a general normal aspect but contained black whorls of membranes (Figure 7Q), multiple abnormal vesicular cristae (left upper inset of Figure6T), or even electron lucent large vesicular cristae (right upper inset of Figure 7T). Plasma membrane of several analyzed cells was interrupted, in such regions the myofibrils being disorganized and the cytoplasm more electron lucent (Figure 7U). A moderate (Figure 7V) or even a very high number (inset of Figure 7V) of transcytosis vesicles were identified in the capillaries.

Concerning other representative ultrastructural features of the myocardium, no noticeable differences were found when compared the junctions forming intercalated discs, the number and aspect of fibroblasts and the amount of intercellular collagen among the four groups.
